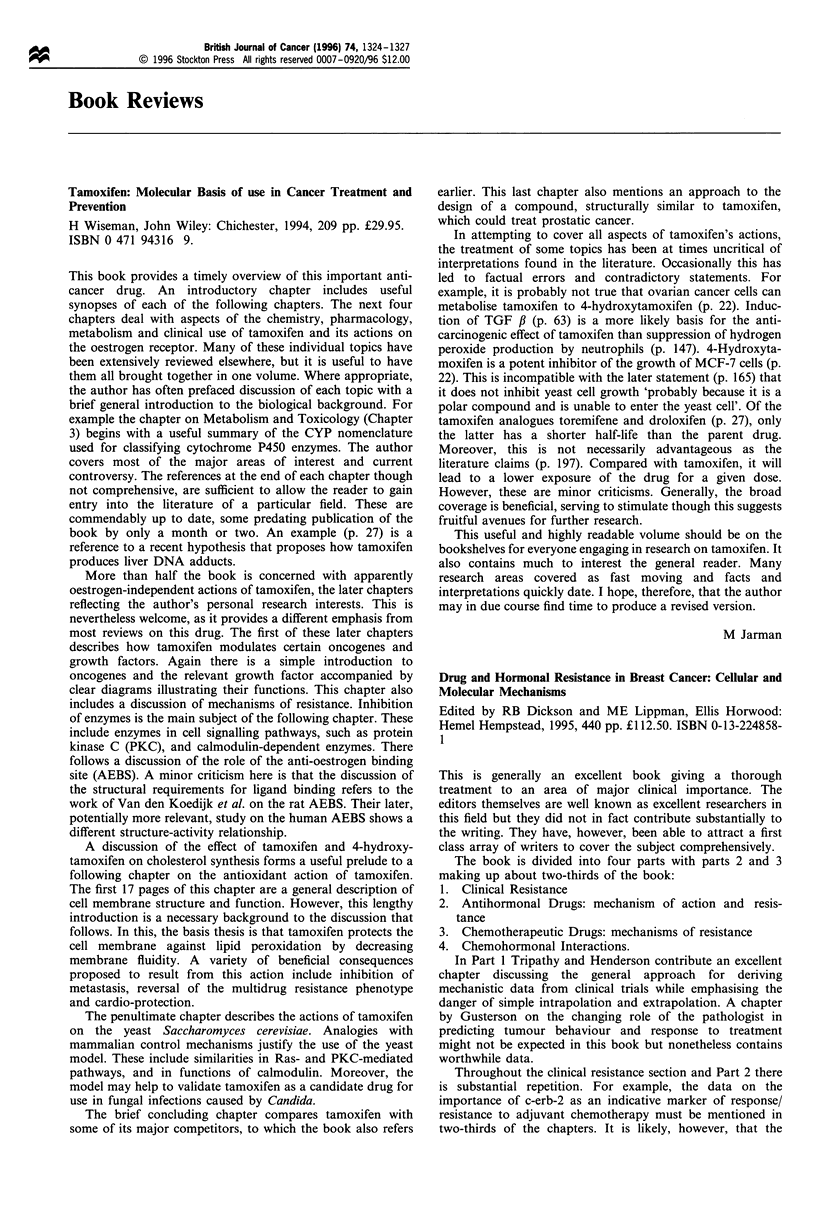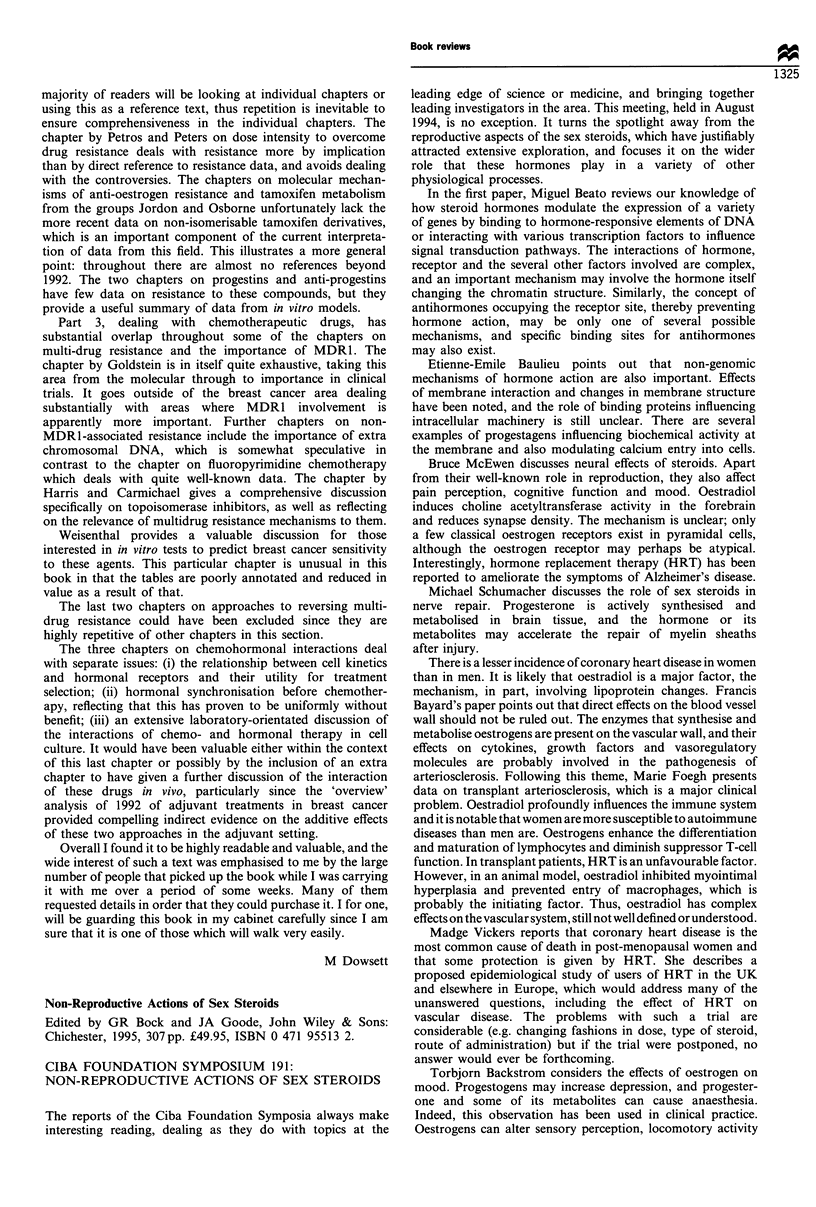# Drug and hormonal resistance in breast cancer: cellular and molecular mechanisms

**Published:** 1996-10

**Authors:** M Dowsett


**Drug and Hormonal Resistance in Breast Cancer: Cellular and Molecular Mechanisms**

Edited by RB Dickson and ME Lippman, Ellis Horwood: Hemel Hempstead, 1995, 440 pp. £112.50. ISBN 0-13-224858-1

This is generally an excellent book giving a thorough treatment to an area of major clinical importance. The editors themselves are well known as excellent researchers in this field but they did not in fact contribute substantially to the writing. They have, however, been able to attract a first class array of writers to cover the subject comprehensively.

The book is divided into four parts with parts 2 and 3 making up about two-thirds of the book:

1. Clinical Resistance
2. Antihormonal Drugs: mechanism of action and resistance
3. Chemotherapeutic Drugs: mechanisms of resistance
4. Chemohormonal Interactions.

In Part 1 Tripathy and Henderson contribute an excellent chapter discussing the general approach for deriving mechanistic data from clinical trials while emphasising the danger of simple intrapolation and extrapolation. A chapter by Gusterson on the changing role of the pathologist in predicting tumour behaviour and response to treatment might not be expected in this book but nonetheless contains worthwhile data.

Throughout the clinical resistance section and Part 2 there is substantial repetition. For example, the data on the importance of c-erb-2 as an indicative marker of response/resistance to adjuvant chemotherapy must be mentioned in two-thirds of the chapters. It is likely, however, that the majority of readers will be looking at individual chapters or using this as a reference text, thus repetition is inevitable to ensure comprehensiveness in the individual chapters. The chapter by Petros and Peters on dose intensity to overcome drug resistance deals with resistance more by implication than by direct reference to resistance data, and avoids dealing with the controversies. The chapters on molecular mechanisms of anti-oestrogen resistance and tamoxifen metabolism from the groups Jordon and Osborne unfortunately lack the more recent data on non-isomerisable tamoxifen derivatives, which is an important component of the current interpretation of data from this field. This illustrates a more general point: throughout there are almost no references beyond 1992. The two chapters on progestins and anti-progestins have few data on resistance to these compounds, but they provide a useful summary of data from *in vitro* models.

Part 3, dealing with chemotherapeutic drugs, has substantial overlap throughout some of the chapters on multi-drug resistance and the importance of MDR1. The chapter by Goldstein is in itself quite exhaustive, taking this area from the molecular through to importance in clinical trials. It goes outside of the breast cancer area dealing substantially with areas where MDR1 involvement is apparently more important. Further chapters on non-MDR1-associated resistance include the importance of extra chromosomal DNA, which is somewhat speculative in contrast to the chapter on fluoropyrimidine chemotherapy which deals with quite well-known data. The chapter by Harris and Carmichael gives a comprehensive discussion specifically on topoisomerase inhibitors, as well as reflecting on the relevance of multidrug resistance mechanisms to them.

Weisenthal provides a valuable discussion for those interested in *in vitro* tests to predict breast cancer sensitivity to these agents. This particular chapter is unusual in this book in that the tables are poorly annotated and reduced in value as a result of that.

The last two chapters on approaches to reversing multi-drug resistance could have been excluded since they are highly repetitive of other chapters in this section.

The three chapters on chemohormonal interactions deal with separate issues: (i) the relationship between cell kinetics and hormonal receptors and their utility for treatment selection; (ii) hormonal synchronisation before chemotherapy, reflecting that this has proven to be uniformly without benefit; (iii) an extensive laboratory-orientated discussion of the interactions of chemo- and hormonal therapy in cell culture. It would have been valuable either within the context of this last chapter or possibly by the inclusion of an extra chapter to have given a further discussion of the interaction of these drugs *in vivo*, particularly since the 'overview' analysis of 1992 of adjuvant treatments in breast cancer provided compelling indirect evidence on the additive effects of these two approaches in the adjuvant setting.

Overall I found it to be highly readable and valuable, and the wide interest of such a text was emphasised to me by the large number of people that picked up the book while I was carrying it with me over a period of some weeks. Many of them requested details in order that they could purchase it. I for one, will be guarding this book in my cabinet carefully since I am sure that it is one of those which will walk very easily.

M Dowsett